# Disparities in hypertensive disorders of pregnancy and severe maternal morbidity: Evidence from a statewide birth cohort

**DOI:** 10.1002/pmf2.70493

**Published:** 2026-10-24

**Authors:** Alyssa Solberg, Kara Hoppe, Ronald Gangnon, Jacqueline M. Powell

**Affiliations:** ^1^ University of Wisconsin School of Medicine and Public Health Madison Wisconsin USA; ^2^ Division of Maternal Fetal Medicine Department of Obstetrics and Gynecology University of Wisconsin Madison School of Medicine and Public Health Madison Wisconsin USA; ^3^ Department of Population Health Sciences Department of Biostatistics and Medical Informatics University of Wisconsin Madison School of Medicine and Public Health Madison Wisconsin USA

**Keywords:** epidemiology, health disparities, hypertensive disorders of pregnancy, preeclampsia, real‐world data, rural health, severe maternal morbidity

## Abstract

**Objective:**

Hypertensive disorders of pregnancy (HDP) complicate 15% of pregnancies nationally and are a major contributor to severe maternal morbidity (SMM). We aimed to characterize temporal trends and demographic disparities in HDP incidence and evaluate the association between HDP and SMM in a statewide cohort.

**Methods:**

This study is a descriptive and regression‐based analysis of a retrospective cohort of Wisconsin births from 2017 to 2023. Data were obtained from the Wisconsin Hospital Association (WHA). Subtypes of HDP were identified using ICD‐10 codes. Incidence rates of HDP per 10,000 births and average annual percent changes (AAPC) were estimated using Poisson regression and examined across demographic variables. Outcomes measured included SMM incidence, AAPCs, and SMM indicators by HDP.

**Results:**

Among 388,250 pregnancies, the incidence of nearly all HDP subtypes rose from 2017 to 2023. Severe pre‐eclampsia had the highest AAPC (11.8%; 95% confidence interval [CI], 10.6%–13.0%). HDP incidence was highest in urban areas, though the AAPC in preeclampsia was greater in rural areas (8.7%; 95% CI, 7.1%–10.3%; *p* = 0.004). Black patients experienced higher incidences of HDP, with significant differences in AAPC for HDP overall, chronic hypertension, and severe preeclampsia (*p* < 0.001). Patients with HDP had higher SMM rates compared to those without, though the AAPC in SMM did not differ by HDP status.

**Conclusion:**

These findings highlight a growing burden of HDP in Wisconsin, with an increase in severe preeclampsia incidence and disparities by geography and race. Targeted interventions in rural areas and for Black patients are needed to improve disparities in HDP incidence and reduce SMM across Wisconsin.

## 1 INTRODUCTION

Hypertensive disorders of pregnancy (HDP) affect 15% of pregnancies in the United States and contribute significantly to maternal morbidity [1]. Chronic hypertension and HDP increase the risk of severe maternal morbidity (SMM), a nationally recognized composite of diagnoses associated with adverse short‐ and long‐term health outcomes, including cardiovascular, metabolic, and renal disease [1, 2, 3, 4, 5].

Although national trends demonstrate rising HDP incidence, state‐level incidence and variation by geography and sociodemographic factors remains poorly characterized. Prior research demonstrates that new‐onset HDP doubled from 2007 to 2019, with the Midwest exhibiting the highest regional incidence and unique rural–urban patterns [6]. However, national estimates rely on data through 2019 and may underestimate current trends. In Wisconsin, preliminary state registry data suggest a substantially higher HDP prevalence (approximately 22%), highlighting the need for state‐level analyses to refine national estimates and identify regional drivers.

Marked racial disparities persist in hypertensive pregnancy outcomes, with Black patients in Wisconsin experiencing substantially higher maternal mortality and disproportionate morbidity related to preeclampsia and eclampsia [7]. Public versus private insurance coverage may also play a role in shaping temporal and geographical trends. Previous studies have linked robust Medicaid coverage to better preventative care and more favorable outcomes among those with hypertension diagnosed in pregnancy [8, 9].

Given the unique temporal and geographic trends in HDP across the Midwest and the confluence of sociodemographic factors, this study aims to characterize contemporary trends in HDP across Wisconsin by geography, race, and insurance status, and to evaluate the contribution of HDP to trends in SMM statewide.

## 2 MATERIALS AND METHODS

We conducted descriptive and regression‐based analyses of a retrospective cohort of in‐hospital births in Wisconsin from March 2017 to December 2023. Data were obtained from the Wisconsin Hospital Association (WHA) and reported to the WHA Information Center (WHAIC) pursuant to Chapter 153 of the Wisconsin Statutes. This dataset included patient demographic characteristics, admission and discharge information, charge and payer data, and diagnostic and procedure codes derived from administrative billing and inpatient discharge records.

To construct our dataset, we included all hospital discharge records with diagnosis‐related group codes corresponding to vaginal and cesarean deliveries during the study period. Demographic variables included self‐identified race and ethnicity, payer type, 5‐digit ZIP code, and county of residence.

Because the dataset lacked patient‐level identifiers, multiple deliveries to the same individual within the study period could not be identified. Therefore, each delivery encounter was treated as an independent birth encounter, and exact duplicate records were excluded. Data were obtained from hospital records within the state of Wisconsin. Therefore, deliveries among Wisconsin residents that occurred outside the state, as well as non‐hospital deliveries, were not included in this cohort. Patient‐level identifiers were not available; therefore, informed consent was not required, and the study was IRB exempt.

The primary outcome was to characterize the incidence of HDP across Wisconsin during the study period. Descriptive analyses were used to examine the incidence of HDP by race and ethnicity, geographic location, and payer status. The secondary outcome was to assess SMM incidence rates and AAPCs by HDP status.

Cases of HDP were identified using ICD‐10 codes and categorized into subtypes including gestational hypertension, chronic hypertension, preeclampsia, preeclampsia with severe features, and HELLP syndrome. The overall HDP category included all encounters meeting ICD‐10 code criteria for any hypertensive disorder of pregnancy. Three non‐specific ICD‐10 categories were excluded from analysis because they could not be assigned to a discrete HDP subtype. These categories represented a small proportion of the overall cohort and were retained in the overall HDP category. The ICD‐10 codes used to define HDP and its subtypes are summarized in Appendix 1.

To characterize temporal trends in the incidence of HDP and its subtypes between 2017 and 2023, we calculated quarterly and annual incidence rates of chronic hypertension, HDP, and its subtypes per 10,000 live births and corresponding 95% confidence intervals using Poisson models. Average annual percent change (AAPC) over the study period was calculated using Poisson regression models.

We conducted descriptive analyses to examine annual HDP incidence rates and AAPCs by urbanization status, race and ethnicity, and insurance type. Urban versus rural residence was defined by county of residence. The Wisconsin Department of Health Services county classification system was used to designate rural versus urban status [10]. Racial and ethnic groups included American Indian/Alaskan Native (AI/AN), Asian/Pacific Islander (Asian/PI), Black, Hispanic, White, and Unknown. All racial and ethnic groups listed were included in analyses of HDP incidence. Black, White, and Hispanic individuals were selected for further discussion because the larger sample size within our cohort and increased representation in the overall Wisconsin population allowed for more robust analyses [11]. Payer categories included private insurance, Medicaid, other government, self‐pay, and unknown. Patients with private insurance and Medicaid comprised a large proportion of the study cohort, with the remaining three categories representing a relatively small number of patients. Therefore, private insurance and Medicaid were selected for further analysis and discussion.

Categorical demographic variables were summarized as frequencies and proportions. Distributions between births with and without HDP were compared using chi‐squared tests. Differences in HDP incidence and AAPCs between demographic groups were calculated using Poisson regression models, with corresponding 95% confidence intervals and *p*‐values.

SMM was defined by the Centers of Disease Control and Prevention (CDC) standard definition and associated ICD‐10 codes, which includes 21 diagnoses [5]. Consistent with prior methodology, blood transfusion was excluded due to its high prevalence [12]. Therefore, the final SMM indicators comprised the remaining 20 diagnostic codes summarized in Appendix 2. These diagnostic indicators were requested during dataset construction, allowing SMM events to be individually linked to patients’ HDP statuses.

Annual SMM incidence rates, both overall and by individual SMM diagnoses, were calculated per 10,000 live births and compared by the presence or absence of HDP. Chi‐squared tests were used to compare incidences of overall SMM and individual SMM diagnoses by HDP status.

## 3 RESULTS

### 3.1 Cohort characteristics

This study included 388,250 deliveries in Wisconsin between 2017 to 2023, of which 17.38% were complicated by HDP. Most births occurred among individuals identifying as White, residing in urban counties, and covered by private insurance (Table 1).

**TABLE 1:** Demographic characteristics of deliveries in Wisconsin, 2017–2023, reported by hypertensive disorder of pregnancy status.

			HDP		
		Overall	No	Yes	
Characteristic	*N*	*N* = 388,250^a^	*N* = 320,783^a^	*N* = 67,467^a^	*p* value^b^
Race/Ethnicity	377,595				<0.001
AI/AN		4539 (1.2%)	3708 (1.2%)	831 (1.3%)	
Asian/PI		19,104 (5.1%)	16,998 (5.5%)	2106 (3.2%)	
Black		45,457 (12%)	33,732 (11%)	11,725 (18%)	
Hispanic		38,002 (10%)	32,676 (10%)	5326 (8.1%)	
White		270,493 (72%)	224,632 (72%)	45,861 (70%)	
Unknown		10,655	9037	1618	
Payer	386,800				<0.001
Medicaid		136,181 (35%)	112,635 (35%)	23,546 (35%)	
Other government		7387 (1.9%)	5965 (1.9%)	1422 (2.1%)	
Private insurance		240,253 (62%)	198,388 (62%)	41,865 (62%)	
Self‐pay		2979 (0.8%)	2555 (0.8%)	424 (0.6%)	
Unknown		1450	1240	210	
Region	388,250				<0.001
Northeastern		83,489 (22%)	71,148 (22%)	12,341 (18%)	
Northern		27,250 (7.0%)	24,016 (7.5%)	3234 (4.8%)	
Southeastern		160,270 (41%)	129,899 (40%)	30,371 (45%)	
Southern		75,302 (19%)	59,928 (19%)	15,374 (23%)	
Western		41,939 (11%)	35,792 (11%)	6147 (9.1%)	
Urban/Rural	388,250				<0.001
Rural		82,731 (21%)	70,618 (22%)	12,113 (18%)	
Urban		305,519 (79%)	250,165 (78%)	55,354 (82%)	
Severe maternal morbidity	388,250	2634 (0.7%)	1322 (0.4%)	1312 (1.9%)	<0.001

Abbreviation: HDP, Hypertensive disorders of pregnancy.

^a^ *n* (%).

^b^ Pearson's Chi‐squared test.

### 3.2 Overall HDP incidence and temporal trends

The incidence of HDP and nearly all subtypes increased from 2017 to 2023, with an overall rise in HDP incidence reflected by a positive AAPC. (Figure 1A). Among HDP subtypes, severe preeclampsia rose the most dramatically with an AAPC of 11.8% (95% confidence interval [CI], 10.6%–13.0%). Conversely, Hemolysis, Elevated Liver enzymes, Low Platelets (HELLP) syndrome was the only subtype with a declining temporal trend (AAPC of −3.5%; 95% CI; −6.8% to −0.1%).

**FIGURE 1:**
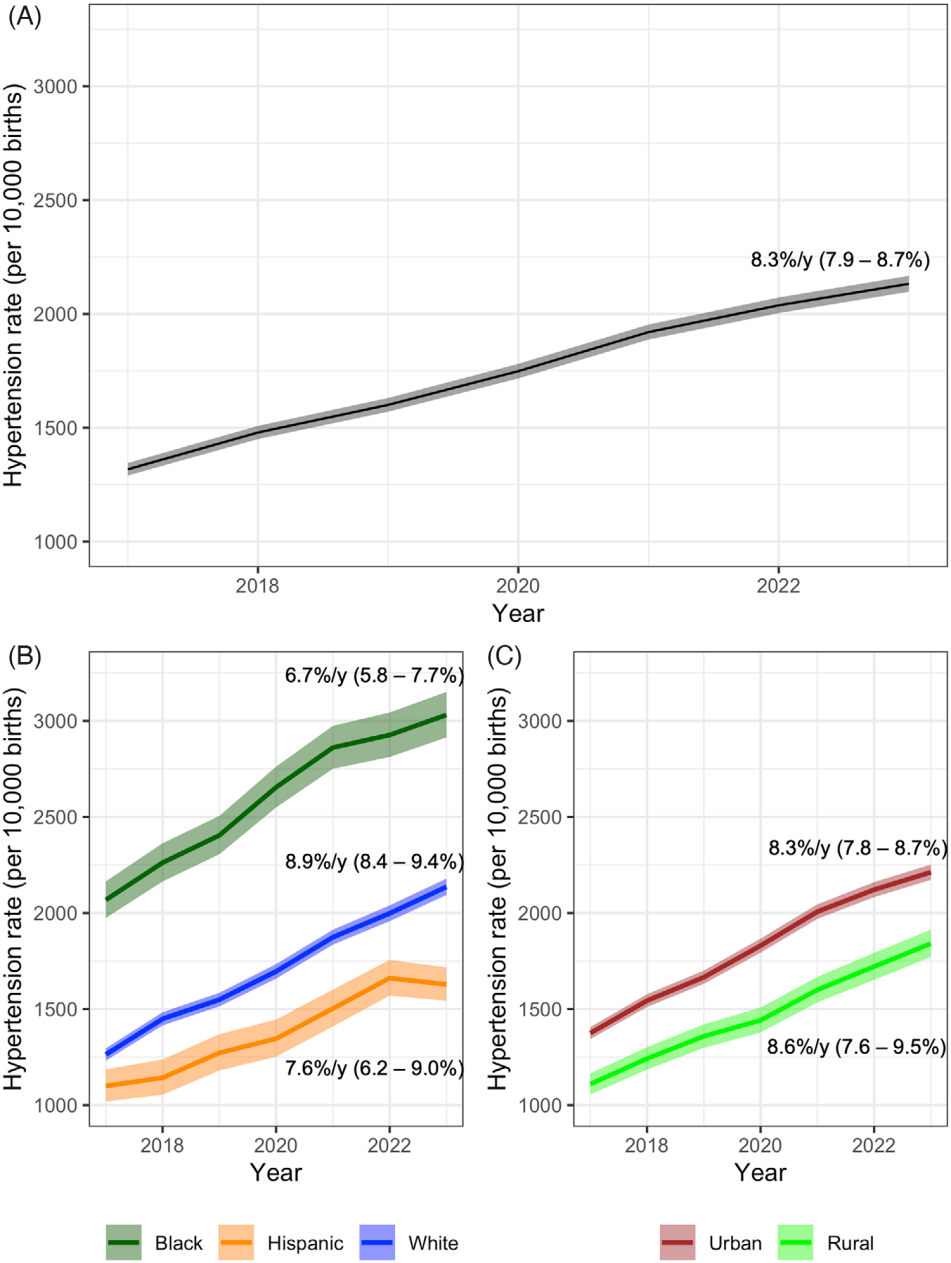
Incidence of hypertensive disorders of pregnancy in Wisconsin per 10,000 births, 2017–2023 (A), by race and ethnicity (B), and urbanization status (C). Average annual percent change estimates are shown for each group. Shaded areas represent 95% confidence intervals.

### 3.3 HDP incidence and temporal trends by race and ethnicity

In addition to overall trends in HDP incidence, we observed notable racial and ethnic disparities. Black patients had the highest incidence of HDP, and nearly all subtypes followed by White and Hispanic patients (Figure 1B). HELLP syndrome was the only exception, with the lowest incidence among Black patients (13.8 cases per 10,000 births; 95% CI, 7.0–27.2) compared with Hispanic patients (14.5; 95% CI, 7.9–26.6) and White patients (21.9; 95% CI, 17.5–27.3) in 2023.

Disparities were also evident in temporal trends. There were significant differences in AAPCs across racial and ethnic groups for HDP overall, chronic hypertension, and severe preeclampsia (*p* < 0.001), and AAPCs for gestational hypertension trended towards significance (*p* = 0.055). White patients demonstrated the highest AAPC in overall HDP, followed by Hispanic and Black patients (Figure 1B).

Incidence of severe preeclampsia increased markedly across all racial and ethnic groups. Among Black and White patients, severe preeclampsia demonstrated the highest AAPC of all HDP subtypes, at 13.3% (95% CI, 11.8%–14.9%) and 8.7% (95% CI, 6%–11.5%) respectively. Among Hispanic patients, gestational hypertension showed the greatest AAPC at 7.7% (95% CI, 6.1 ‐ 9.3%).

### 3.4 HDP incidence and temporal trends by urbanization status

We next investigated geographic differences in HDP incidence, comparing outcomes by urbanization status. Across the study period, HDP incidence rose in both urban and rural settings (Figure 1C). Patients in urban areas experienced higher incidences of HDP and nearly all subtypes, excluding HELLP syndrome. However, the incidence of preeclampsia increased sharply in rural compared to urban areas with an AAPC of 8.7% (95% CI, 7.1 ‐ 10.3%, *p* = 0.004).

Differences in the AAPC between urban and rural settings were not statistically significant for overall HDP, gestational hypertension, chronic hypertension, severe preeclampsia, and HELLP syndrome. Nevertheless, AAPCs tended to be higher in rural areas compared to urban areas for all disease subtypes aside from HELLP syndrome.

Severe preeclampsia exhibited the most pronounced rise across the study period in both settings, with AAPCs of 13.5% in rural areas (95% CI, 10.6%–16.4%) and 11.5% in urban areas (95% CI, 10.1%–12.8%), though this difference was not statistically significant (*p* = 0.213)

### 3.5 HDP incidence and temporal trends by payer status

When calculated by insurance type, HDP incidence increased from 2017 to 2023 among both privately and publicly insured patients. In publicly insured patients, HDP incidence rose from 1297.2 (95% CI, 1253.0–1342.8) to 2059.4 (95% CI, 2002.0–2118.0) cases per 10,000 births. Among privately insured patients, incidence rose from 1323.4 (95% CI, 1288.7–1358.8) to 2181.7 (95% CI, 2137.5–2226.8) cases per 10,000 births. There was no significant difference in AAPCs between groups (*p* = 0.227).

Incidence of severe preeclampsia rose the most drastically across both groups with AAPCs of 12.5% (95% CI, 10.9%–14.1%) among privately insured patients and 11.4% (95% CI, 9.4%–13.4%) among publicly insured patients, though this difference was not statistically significant (*p* = 0.385).

### 3.6 Severe maternal morbidity by HDP status

Our secondary outcome was to analyze the relationship between HDP and SMM across the study period. SMM was significantly more common among patients with an HDP diagnosis, with incidence approximately 4 to 5 times higher from 2017 to 2023 compared with patients without HDP (Table 2). Among HDP subtypes, those with HELLP syndrome experienced the highest SMM, although this subgroup was small. Despite absolute differences in SMM incidence, the AAPC in SMM did not differ significantly by HDP status, with AAPCs of 3.5% (95% CI, 0.7%–6.4%) among those with HDP and 4.9% (95% CI, 2.1%–7.7%) among those without HDP (*p* = 0.508).

**TABLE 2:** Comparison of severe maternal morbidity overall and by individual indicator among Wisconsin deliveries with and without hypertensive disorders of pregnancy, 2017–2023.

		HDP		
		No	Yes	
Characteristic	Overall *N* = 388,250	*N* = 320,783^a^	*N* = 67,467^a^	*p* value^b^
Severe maternal morbidity	2634 (0.7%)	1322 (0.4%)	1312 (1.9%)	<0.001
Acute renal failure	625 (0.2%)	134 (<0.1%)	491 (0.7%)	<0.001
Disseminated intravascular coagulation	652 (0.2%)	408 (0.1%)	244 (0.4%)	<0.001
Adult respiratory distress syndrome	373 (<0.1%)	181 (<0.1%)	192 (0.3%)	<0.001
Eclampsia	185 (<0.1%)	0 (0%)	185 (0.3%)	<0.001
Pulmonary edema/acute heart failure	179 (<0.1%)	38 (<0.1%)	141 (0.2%)	<0.001
Hysterectomy	414 (0.1%)	295 (<0.1%)	119 (0.2%)	<0.001
Sepsis	293 (<0.1%)	198 (<0.1%)	95 (0.1%)	<0.001
Shock	253 (<0.1%)	160 (<0.1%)	93 (0.1%)	<0.001
Ventilation	161 (<0.1%)	86 (<0.1%)	75 (0.1%)	<0.001
Puerperal cerebrovascular disorders	139 (<0.1%)	70 (<0.1%)	69 (0.1%)	<0.001
Air and thrombotic embolism	124 (<0.1%)	85 (<0.1%)	39 (<0.1%)	<0.001
Conversion of cardiac rhythm	35 (<0.1%)	15 (<0.1%)	20 (<0.1%)	<0.001
Acute myocardial infarction	24 (<0.1%)	9 (<0.1%)	15 (<0.1%)	<0.001
Cardiac arrest/ventricular fibrillation	28 (<0.1%)	16 (<0.1%)	12 (<0.1%)	<0.001
Sickle cell disease with crisis	22 (<0.1%)	12 (<0.1%)	10 (<0.1%)	0.001
Aneurysm	26 (<0.1%)	17 (<0.1%)	9 (<0.1%)	0.039
Severe anesthesia complications	28 (<0.1%)	21 (<0.1%)	7 (<0.1%)	0.4
Heart failure/arrest during surgery or procedure	5 (<0.1%)	1 (<0.1%)	4 (<0.1%)	0.002
Amniotic fluid embolism	21 (<0.1%)	17 (<0.1%)	4 (<0.1%)	>0.9

Abbreviation: HDP, Hypertensive disorders of pregnancy.

^a^ *n* (%).

^b^ Pearson's Chi‐squared test.

Several SMM indicators occurred significantly more often in patients with HDP. The most frequent complications among patients with an HDP diagnosis included acute renal failure, disseminated intravascular coagulation, and adult respiratory distress syndrome. Of the 20 indicators analyzed, only amniotic fluid embolism and severe anesthesia complications did not differ significantly between groups (Table 2). Notably, these complications were extremely rare, affecting fewer than 30 patients out of the cohort.

## 4 DISCUSSION

In 2023, more than one in five births (21%) in Wisconsin was complicated by HDP, compared with approximately one in eight (13%) in 2017. While estimates vary, incidence in Wisconsin exceeds the national average and mirrors broader temporal patterns reported in the literature [1, 6, 13, 14, 15]. While national HDP incidence increased annually by 7.3% from 2011 to 2019, we observed a steeper rise in Wisconsin (AAPC 8.3%), providing state‐specific context and evidence of the growing burden of HDP [14].

Severe preeclampsia increased substantially over the study period, consistent with prior reports [16, 17]. Earlier studies demonstrated a 6.7‐fold increase in the risk of developing preeclampsia between 1980 and 2003; however, these data predated the 2013 ACOG revision eliminating proteinuria as a diagnostic requirement [23]. Consequently, our more recent estimates may better reflect contemporary disease burden.

After stratifying our results by race and ethnicity, we observed disparities in HDP incidence and AAPC. In 2023, Black patients, experienced HDP at nearly double the rate of Hispanic patients and over 40% more than White patients. Despite the faster relative rise among Hispanic and White patients, the absolute burden remains disproportionately high in the Black population. These findings reflect the enduring impact of structural racism and systemic inequities that shape health outcomes across the lifespan [18, 19, 20]. Future studies should examine how neighborhood‐level health care access and cumulative stress exposures contribute to observed disparities.

Across the study period, urban HDP incidence exceeded that of rural areas. In 2023, urban regions had 20% more cases per 10,000 births. However, rural areas exhibited higher AAPCs across most HDP categories, reaching significance for preeclampsia. This may suggest rural incidence is converging with urban trends. Our findings parallel Midwest‐specific evidence of higher urban HDP incidence [6]. Disparities may reflect differences in environmental risk factors and access to obstetric care, which has declined substantially in rural Wisconsin in the past decade [21]. Further research is needed to assess how rural‐specific environmental exposures and declining access to care contribute to these trends.

The secondary outcome demonstrated that patients with an HDP diagnosis were five times more likely to develop SMM compared to those without. Despite this difference in incidence, the AAPC in SMM did not differ significantly based on HDP status. This may be influenced by the rising rate of SMM in the general population, which has been attributed to the rising age and growing number of comorbidities among those of reproductive age [22, 23, 24]. This finding could also reflect advancements in management of HDP and increased awareness and use of prophylactic low‐dose aspirin among individuals at risk [25, 26, 27].

Low‐dose aspirin was recommended in 2013 for prevention of preeclampsia in high‐risk individuals, yet uptake in the United States remains low despite strong recommendations from the US Preventative Services Task Force (USPSTF), the American College of Obstetricians and Gynecologists (ACOG), and the Society for Maternal‐Fetal Medicine (SMFM) [28]. Fewer than 50% of high‐risk and fewer than 25% of moderate‐risk patients use aspirin for prevention. This underuse is concerning given rising rates of hypertensive disorders of pregnancy in Wisconsin.

A University of Wisconsin‐Madison quality improvement project improved documented aspirin recommendations from 28% to 79% after implementing universal screening [29]. Future work should evaluate statewide aspirin use to identify geographic gaps and guide targeted interventions to increase utilization and reduce severe preeclampsia incidence.

The most common SMM indicators among HDP patients mirrored statewide data, with one exception [22]. Hysterectomy was more prevalent in the general population compared to our cohort. Our findings largely reflect the underlying pathophysiology of the disease process. HDP is characterized by abnormal placentation and systemic vascular endothelial dysfunction resulting in an inflammatory cascade that increases risk for complications like acute renal failure and cardiovascular morbidity [30]. These morbidity patterns validate the distinct risk profile associated with HDP and provide a benchmark to evaluate the effectiveness of targeted interventions.

This study has limitations, including the possibility of miscoding errors. The WHA database is comprised of ICD‐10 codes. Therefore, accuracy relies on provider coding practices. However, ICD‐10 codes are a commonly used and validated metric for evaluating disease incidence in retrospective cohorts, with relatively high specificity and positive predictive value for identifying HDP [31, 32].

Regarding cohort selection, this dataset did not capture deliveries among Wisconsin residents that occurred outside the state. Non‐hospital deliveries were also excluded. Selection bias may have been introduced if the demographics and discharge diagnoses of excluded individuals differed systematically from those included in the study cohort. Demographic comparisons and incidence estimates may be affected by this bias. Furthermore, urbanization status in this study was defined at the county level. More granular geographic classification may have better captured within‐county variation, potentially yielding more accurate incidence and AAPC trends by urbanization status.

Additionally, our dataset only captured ICD‐10 codes present at discharge, potentially leading to an underestimation of postpartum HDP incidence. The rarity of certain SMM indicators also led to low sample size and thus may not reflect the true distribution of these SMM indicators among individuals with HDP.

Despite these limitations, our study had many strengths. The WHA dataset included a large proportion of births in Wisconsin, capturing approximately 380,000 births across the study period. This robust sample allowed analysis of trends that may be applicable to populations beyond Wisconsin. Furthermore, this dataset, which reported SMM events for each individual birth encounter, allowed us to examine the true burden of HDP and broader maternal morbidity in this statewide cohort.

## 5 CONCLUSION

In this retrospective cohort of over 380,000 deliveries in Wisconsin from 2017 to 2023, we observed a marked increase in HDP incidence across all populations, with a steep rise in severe preeclampsia and pronounced demographic and geographic disparities. HDP was significantly associated with higher SMM risk, with key indicators accounting for a substantial proportion of SMM cases. Importantly, this analysis likely represents the most up‐to‐date state‐level estimates of HDP incidence, which may reflect ongoing annual rise not yet captured in national surveillance. This underscores the value of timely state‐level data to inform awareness, targeted interventions to educate providers and patients across Wisconsin, quality improvement initiatives to lower the incidence of HDP, and future research efforts to identify drivers of HDP across Wisconsin, (see Supporting Information).

## CONFLICT OF INTEREST STATEMENT

The authors declare no conflicts of interest.

## Supporting information



Supporting information

## APPENDIX 1 International statistical classification of diseases and related health problems, 10th revision codes used to define hypertensive disorders of pregnancy and its subtypes in the Wisconsin Hospital Association dataset

### 1.1

**Table:**

Diagnosis	ICD‐10 codes
Hypertensive disorders of pregnancy	O10.01, O10.02, O10.12, O10.22, O10.42, O10.91, O10.92, O11.3, O11.4, O11.5, O12.04, O12.05, O12.13, O12.14, O12.24, O13.3, O13.4, O13.5, O13.9, O.14, O14.03, O14.04, O14.05, O14.1, O14.13, O14.14, O14.15, O14.23, O14.24, O14.25, O14.90, O14.93, O14.94, O14.95, O15.1, O15.2, O16.3, O16.4
Chronic hypertension	O10.01, O10.02, O10.12, O10.22, O10.42, O10.91, O10.92
Gestational hypertension	O13.3, O13.4, O13.5, O13.9
Preeclampsia	O.14, O14.03, O14.04, O14.05
Preeclampsia with severe features	O14.1, O14.13, O14.14, O14.15
HELLP syndrome	O14.23, O14.24, O14.25

Abbreviation: HELLP, Hemolysis, Elevated Liver enzymes, Low Platelets.

## APPENDIX 2 International statistical classification of diseases and related health problems, 10th revision codes used to define severe maternal morbidity in the Wisconsin Hospital Association dataset

### 2.1

**Table:**

Diagnosis	ICD‐10 codes
Acute myocardial infarction	121.xx and 122.x
Aneurysm	171.xx and 179.0
Acute renal failure	N17.x and O90.4
Acute respiratory distress syndrome	J80, J95.1, J95.2, J95.3, J95.82x, J96.0x, J96.2x, J96.9x, R06.03, R09.2
Amniotic fluid embolism	O88.112, O88.113, O88.119, O88.12, O88.13
Cardiac arrest/ventricular fibrillation	I46.x, I49.0x
Conversion of cardiac rhythm	5A12012, 5A2204Z
Disseminated intravascular coagulation	D65, D68.8, D68.9, O45.002, O45.003, O45.009, O45.012, O45.013, O45.019, O45.022, O45.023, O45.029, O45.092, O45.093, O45.099, O46.002, O46.003, O46.009, O46.012, O46.013, O46.019, O46.022, O46.023, O46.029, O46.092, O46.093, O46.099, O67.0, O72.3
Eclampsia	O15.X
Heart failure/arrest during surgery or procedure	I97.120, I97.121, I97.130, I97.131, I97.710, I97.711
Puerperal cerebrovascular disorders	A81.2, G45.x, G46.x, G93.49, H34.0x, I60.xx, I61.xx, I62.xx, I63.00, I63.01x, I63.1xx, I63.2xx, I63.3xx, I63.4xx, I63.5xx, I63.6, I63.8x, I63.9, I65.xx, I66.xx, I67.xx, I68.xx, O22.50, O22.52, O22.53, I97.810, I97.811, I97.820, I97.821, O87.3
Pulmonary edema/acute heart failure	I50.1, I50.20, I50.21, I50.23, I50.30, I50.31, I50.33, I50.40, I50.41, I50.43, I50.810, I50.811, I50.813, I50.814, I50.82, I50.83, I50.84, I50.89, I50.9, J81.0
Severe anesthesia complications	O29.112–O29.119, O29.122–O29.129, O29.192–O29.199, O29.212–O29.219, O29.292–O29.299, O74.0, O74.1, O74.2, O74.3, O89.0x, O89.1, O89.2, T88.2XXA, T88.3XXA
Sepsis	A32.7, A40.x, A41.x, I76, O85, O86.04, R65.20, R65.21, T81.12XA, T81.44XA
Shock	O75.1, R57.x, T78.2XXA, T81.10XA, T81.11XA, T81.19XA, T88.6XXA
Sickle cell disease with crisis	D57.00, D57.01, D57.02, D57.211, D57.212, D57.219, D57.411, D57.412, D57.419, D57.811, D57.812, D57.819
Air and thrombotic embolism	I26.x, O88.012–O88.03, O88.212–O88.23, O88.312–O88.33, O88.812–O88.83, T80.0XXA
Hysterectomy	0UT90ZL, 0UT90ZZ, 0UT97ZL, 0UT97ZZ
Temporary tracheostomy	0B110F4, 0B113F4, 0B114F4
Ventilation	5A1935Z, 5A1945Z, 5A1955Z
