# Supplementary material for: Disparities in hypertensive disorders of pregnancy and severe maternal morbidity: Evidence from a statewide birth cohort
Source: Pregnancy (Hoboken). 2026 Oct 24;2(6):e70493. doi: 10.1002/pmf2.70493 (PMC13695025; doi:10.1002/pmf2.70493)
Supplement: Supplementary file 1 [file PMF2-2-e70493-s001.docx]

**Supplemental Table 1**

**Supplemental Table 2**

**Supplemental Table 3**

**Supplemental Table 4**

**Supplemental Table 5**
